# DiaBar: Predicting type 2 diabetes remission post-metabolic surgery utilizing mRNA expression profiles from subcutaneous adipose tissue

**DOI:** 10.1016/j.jcte.2025.100410

**Published:** 2025-07-22

**Authors:** Jonas Wagner, Manfred Wischnewsky, Patricia von Kroge, Helge Wilhelm Thies, Pia Roser, Stefan Wolter, Thilo Hackert, Jakob Izbicki, Oliver Mann, Anna Duprée

**Affiliations:** a Department of General-, Visceral- and Thoracic Surgery, University Medical Center Hamburg-Eppendorf, Martinistr. 52, 20246 Hamburg, Germany; b Mathematics and Informatics, University of Bremen, Universitätsallee, 28359 Bremen, Germany; c Department of Gynecology, University Medical Center Hamburg-Eppendorf, Martinistr. 52, 20246 Hamburg, Germany; d Lipocyte BioMed GmbH, Erbrichterweg 7a, 28357 Bremen, Germany; e III. Department of Medicine, University Medical Center Hamburg-Eppendorf, Martinistr. 52, 20246 Hamburg, Germany; f Department of General- and Visceral Surgery, Regio Kliniken, Ramskamp 71-75, 25337 Elmshorn, Germany

**Keywords:** Type 2 diabetes, Metabolic surgery, Remission, Prediction, Subcutaneous adipose tissue

## Abstract

**Background:**

Subcutaneous adipose tissue (SAT) is a metabolic organ, which is involved in the pathogenesis of type 2 diabetes (T2D). Methods to predict diabetes remission after metabolic surgery exist, however their prediction accuracy still needs improvement. We hypothesized, that gene expression profiles in the SAT could predict diabetes remission after metabolic surgery more accurately than any current methods.

**Methods:**

In this retrospective cohort study, we identified individuals who underwent metabolic surgery. We collected SAT biopsies during the surgery and analyzed the expression of *HMGA2*, *PPARG*, *ADIPOQ* and, *IL6*. The American Diabetes Association criteria were used to define partial and complete remission. Univariate generalized linear models, tree decision algorithms (Exhausted Chaid, CART and Quinlan’s C5 with adaptive boosting) and, multilayer perceptron networks were used to develop classifiers for patients with no, partial or complete remission (DiaBar).

**Results:**

In this study 106 patients were included, 66 (62.3%) patients had T2D the remaining 40 (37.7%) were patients with prediabetes. Complete and partial remission were achieved by 69 (65.1%) and 20 (18.9%) patients respectively. Using a multilayer perceptron, we achieved an overall accuracy of 98.0% (remission: no 100%; partial 90.0%; complete 100%). The validated DiaRem Score was used as the comparative score, which had an overall accuracy for classifying patients with complete, partial or no remission of 74.7%.

**Conclusions:**

Using gene expression profiles from the SAT, we developed the DiaBar test, which accurately predicts diabetes remission after metabolic surgery and seems to be superior to the DiaRem score.

## Background

The global prevalence of diabetes is approximately 9.3 %, while type 2 diabetes (T2D) accounts for 90 % of the cases [1]. Using these numbers, it’s estimated that almost 420 million people suffer from T2D worldwide and these numbers are expected to increase in the future [1]. T2D can have fateful consequences, for example T2D is an independent risk factor for cardiovascular disease (CVD) [2]. Obesity is directly linked to T2D and rising prevalence of T2D can be attributed to the rising prevalence of obesity [1,3].

Metabolic surgery has been proven to be the most effective therapy regarding weight loss and T2D. About 30–60 % of patients achieve diabetes remission after metabolic surgery [[4], [5], [6]]. Some factors, which are associated with diabetes remission have already been identified. Early diabetes stage, no use of insulin, younger age and achieving a total body weight loss of more than 20 % after surgery are all known predictors for diabetes remission [[7], [8], [9], [10], [11], [12]]. Conversely, low weight loss, the use of insulin and poorly controlled T2D (HbA1c > 7 %) are negative predictors of diabetes remission [13,14]. Identifying individuals, who will benefit of metabolic surgery, is important in order to help patients and health care providers to make informed decisions. However, identifying patients who achieve remission remains a challenge. Several prediction models already have been proposed, two of these prediction models, the ABCD score and the DiaRem score, were widely externally validated in different populations, in a variety of bariatric procedures, and for both short- and long-term diabetes remission [15]. The DiaRem score has moderate to high discriminant ability (AUC > 0.80) [11,15]. Unfortunately, these tests do not perform well for patients with insulin therapy. Hence, new more accurate tests are needed.

The defining characteristic for obesity is an abnormal or excessive accumulation of fat. This fat can be stored in the subcutaneous tissue [16]. The adipose tissue has recently been focused by researchers as a key component in the development of T2D [17]. Under physiological conditions the white adipose tissue (WAT) influences a variety of task from energy metabolism to inflammation and can be considered as an endocrine organ [18]. During obesity the WAT becomes dysfunctional, leading to local hypoxia, fibrosis and inflammation, which can lead to T2D [19].

Several markers within the adipose tissue have been identified to be involved in the development of T2D. Adiponectin, which is encoded by the *ADIPOQ* gene, appears to be one important factor. For example, overexpression of *ADIPOQ* in ob/ob mice protects against diet induced insulin resistance [20]. Furthermore, adiponectin can be linked to the transcription factor peroxisome proliferator-activated receptor gamma (PPARγ), encoded by *PPARG*[21]. PPARγ is highly expressed in the adipose tissue and was originally identified as an induced factor during adipogenesis [22]. Additionally, it has been identified to be involved in lipid metabolism and glucose homeostasis [23]. Another important transcription factor is high mobility group protein AT-hook 2 (*HMGA2*). *HMGA2* expression in the subcutaneous adipose tissue (SAT) in patients with T2D was elevated compared to patients without T2D [24]. Furthermore, obesity and T2D are associated with a chronic low-grade inflammation [25]. One proinflammatory factor, which is produced by the adipose tissue, is interleukin-6 (*IL6*) and it is implicated in the etiology of T2D [26].

In this single-cohort pilot study the combined role of the biomarkers *HMGA2*, *PPARG*, *ADIPOQ* and *IL6* as predictors for T2D remission after metabolic surgery were evaluated. The connection between HbA1c 12 months after surgery and T2D remissions respectively and, preoperative HbA1c, body mass index (BMI), insulin dependency and the biomarkers *HMGA2*, *PPARG*, *ADIPOQ* and *IL6* were examined.

## Methods

### Participants

Patients with type 2 diabetes mellitus or prediabetes, who underwent metabolic surgery and simultaneous SAT biopsy in our institution were identified. All included patients agreed and signed informed consent. Certified bariatric surgeons performed all surgical procedures in accordance with the German Guidelines of Surgical Treatment of Obesity [27]. Patients were routinely evaluated by a multidisciplinary team before surgery, and we collected data on height, weight, BMI, comorbidities, type of surgery, and standard laboratory parameters. The American Diabetes Association (ADA) guidelines to define type 2 diabetes [28] as well as diabetes remission were used [29]. Type 2 diabetes was accordingly defined as HbA1c above 6.5 % or 48 mmol/ml. Antidiabetic drugs were discontinued in all patients post-surgery. Complete remission was defined as HbA1c below 5.7 % or 38.8 mmol/mol 12 months after surgery, partial remission was defined as HbA1c between 5.7–6.5 % or 38.8–48 mmol/ml 12 month after surgery. Our primary outcome measures were complete, partial or no remission 12 month after surgery.

### Real time polymerase chain reaction (qPCR)

SAT biopsies from patients with obesity and T2D were snap frozen in liquid nitrogen within minutes after surgery and stored at −80 °C until further analysis. To isolate total ribonucleic acid (RNA) from whole tissues samples, we first homogenized pieces of maximum 100 mg using a gentleMACS™ dissociator (Miltenyi, Bergisch Gladbach, Germany) in 1 ml Qiazol (Qiagen, Hilden, Germany). Next, we isolated RNA using the RNeasy Lipid Tissue Mini Kit (Qiagen, Hilden, Germany) according to manufacturer’s protocol. In brief, the samples were transferred in 2 ml tubes and incubated them 5 min (min) at room temperature. Subsequently, Chloroform was then added and the samples were incubated for a further 2–3 min at room temperature. The samples were then centrifuged at 12,000 g for 15 min at 4 °C. Total RNA from the aqueous phase was then transferred into a new tube and loaded onto a QiaCube connect (Qiagen, Hilden, Germany) for further RNA clean up. RNA concentration was measured and up to 250 ng total RNA were reverse transcribed with 200 U of Invitrogen M−MLV reverse transcriptase, Invitrogen RNase OUT and 150 ng Invitrogen random primers (Thermo Fisher Scientific, Karlsruhe, Germany) into complementary deoxyribonucleic acid (cDNA). RNA was denatured at 65 °C for 5 min and subsequently kept on ice for 1 min. After adding the enzyme to the RNA primer mixes, samples were incubated at 25 °C for 10 min to allow annealing of the random primers. Reverse transcription was carried out at 37 °C for 50 min followed by inactivation of the reverse transcriptase at 70 °C for 15 min. A gene-specific preamplification was performed using the PerfeCTa PreAmp 5X SuperMix (Quantabio, Beverly, MA, USA) with the primers given in table S1. The initial denaturation of the cDNA at 95 °C for 2 min was followed by 14 amplification cycles at 95 °C for 10 sec and 60 °C for 3 min. Finally, we performed qPCR using the comparative Ct method (ΔΔCt) with *HPRT* as endogenous control. Quantitative real-time RT-PCR was performed with the FastStart Universal Probe Master ROX (Roche Diagnostics, Mannheim, Germany) on the Applied Biosystems StepOnePlus Real-Time PCR System (Thermo Fisher Scientific, Karlsruhe, Germany). Reaction conditions were 95 °C for 10 min followed by 50 cycles at 95 °C for 15 sec and 60 °C for 1 min. Commercially available gene expression assays were used to quantify the relative expression of *HMGA2* (assay ID Hs00171569_m1), *PPARG* (Hs01115513_m1), *ADIPOQ* (Hs00605917_m1) and *IL6* (Hs00985639_m1; Thermo Fisher Scientific, Karlsruhe, Germany). All reactions were done in triplicate.

### Statistical analysis

Nominal values are described by their absolute and relative frequencies. Continuous variables are presented as mean with standard deviation (SD) or median with interquartile range (IQR) for normally and non-normally distributed variables respectively. The Wilcoxon-Mann-Whitney test as a parameter-free statistical test was used to test the significance of the agreement of distributions. The Kolmogorov-Smirnov test was used to examine the normal distributions of various biomarkers. Univariate generalized linear models (GLMs) were used to reduce within-group error variance and remove confounding factors. To test the assumptions for GLMs, Levene's test for homogeneity of variances was performed. Model goodness and effect sizes (partial η2 and Cohen's d) were calculated for each factor and covariate. Discrimination was evaluated by area under the receiver operating characteristic curve (AUC), a measure of the quality of the test, whereas calibration by the Hosmer–Lemeshow test and predicted versus observed remission ratio. Tree decision algorithms (Exhausted Chaid (Chi-Squared Automatic Interaction Detector)), Classification and Regression Trees (CART) and C5 were used to develop classifiers. In addition, we used a multilayer perceptron network (MLP). A MLP is a feedforward, supervised learning artificial neural network model that maps sets of input data onto a set of appropriate outputs (T2D remission). An MLP consists of multiple layers of nodes in a directed graph, with each layer fully connected to the next one. Except for the input nodes, each node is a processing element (neuron) with a nonlinear activation function. MLPs solve problems stochastically, which provides an approximation of solutions to complex problems. MLPs are universal function approximators, meaning they can model functional relationships (such as the relationship between diagnostic factors and outcome as a function) with arbitrary accuracy. MLP utilizes backpropagation as supervised learning technique for training the network. Hyperbolic tangent (tanh(x)=(e^x^ – e^-x^)/(e^x^ + e^-x^)) was applied as nonlinear activation function for nodes in the hidden layers and softmax as nonlinear activation functions for nodes in the output layer. Softmax converts a vector of numbers into a vector of probabilities and is defined as follows: $\sigma \left(o_{i}\right)=\frac{e^{o_{i}}}{\sum_{j=1}^{n}e^{o_{i}}}$, where the index i is in (0, …, n-1) and o is the output vector of the network.

Cross entropy was used as the error function. Furthermore, we calculated importance and normalized importance for each independent variable (predictor). The importance is a measure of how much the predicted value of the model changes for diverse value of the predictors. It has several purposes. The first being model simplification, variables that do not influence a model’s predictions, may be excluded from the model. The second purpose is domain-knowledge-based model validation. Identification of the most important variables may be helpful in assessing the validity of the model based on domain knowledge. The last purpose is model exploration. Comparison of variables’ importance in different models may help in discovering interrelations between the variables.

A predicted by observed chart displays clustered boxplots of predicted pseudo-probabilities for the categorical dependent variable T2D remission. Statistical significance was defined as p < 0.05. Statistical analysis was performed using R version 4.1 (R Foundation) and IBM SPSS Statistics, version 28.0 (IBM Corp., Armonk, NY, USA).

## Results

### Characterization of the study population (Table 1)

**Fig. 1:**
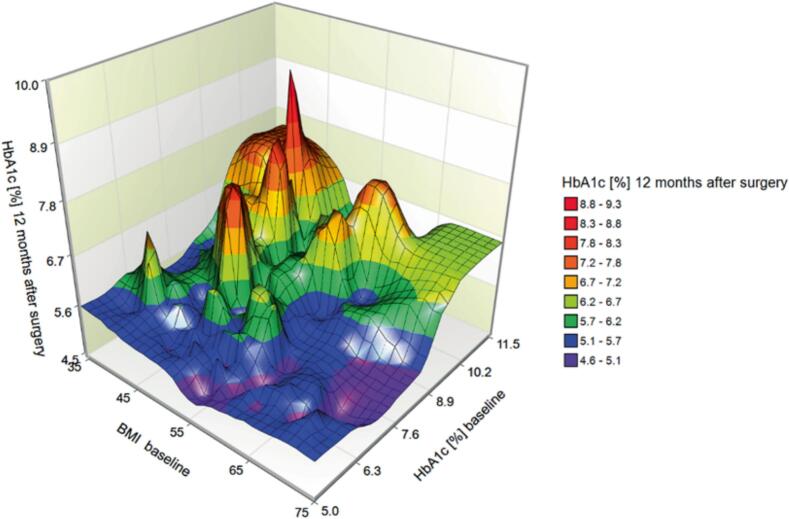
Surface plots depicting the relationship between baseline HbA1c, HbA1c 12 months after surgery and BMI.

A total of 106 patients were included in the study. The patients were mainly female (71.7 %), with a mean BMI of 52–9 ± 8.3 kg/m^2^ and a mean age of 46 ± 10.1 years. We performed sleeve gastrectomy (71.7 %) and Roux-en-Y Gastric Bypass (RYGB) (28.3 %). The mean HbA1c concentration was 7.0 ± 1.5 %, 40.6 % of the patients took at least one diabetes medication, and 35.8 % of the patients with T2DM needed insulin therapy (Table 1). Bariatric surgery resulted in a significant weight loss and an improvement of most laboratory values (Table 1). However, no significant differences were found between sleeve gastrectomy and Roux-en-Y Gastric bypass surgery in terms of the reduction in BMI and the reduction in mean HbA1c levels after a period of 12 months (Sup. Fig. 1 and Sup. Fig. 2). Complete and partial remission at one year were achieved in 66.3 % and 19.8 % of patients respectively (Table 2). Preoperative HbA1c concentrations are significant covariate for HbA1c concentrations 12 months after surgery (p < 0.001). More than one third of the 12 months outcome were determined by preoperative HbA1c concentrations (partial η2 = 0.347; p < 0.0.001). The BMI is another significant predictor for HbA1c at 12 months (p = 0.032). The 3D surface plot with the predictors preoperative HbA1c and BMI shows that patients with preoperative HbA1c ≤ 7 % and HbA1c > 7 % appear to have different outcomes for HbA1c at 12 months. For patients with preoperative HbA1c ≤ 7 %, HbA1c values at 12 months ranged from 4.6 − 6.2 %. Hereby, postoperative HbA1c is inversely related to preoperative BMI. Patients with a preoperative HbA1c ≥ 7 % (n = 38) have HbA1c values at 12 months in the range of 4.8 – 9.6 % with mean 6.4 ± 1.0 (Fig. 1). Preoperative insulin therapy is another significant predictor of HbA1c at 12 months. Approximately 45 % of HbA1c concentrations 12 months after surgery are univariately determined by insulin therapy (partial η^2^ = 0.446; p < 0.0.001). Mean HbA1c 12 months after surgery were 6.8 (95 % CI 6.5–––7.1) for patients with insulin therapy and 5.4 (95 % CI 5.3–––5.6) for patients without. In our data 98 % of patients without insulin use achieved partial or complete remission. Accurately predicting remission rates in patients with insulin therapy is challenging.

**Table 1:** Characterization of the study population.

	Total cohort			Patients with T2DM (baseline)		
	Baseline	12 months postoperative	two sided p-value	Baseline	12 months postoperative	two sided p-value
n	106	101		66	62	
Women [%]	71.7	73.3		68.7	71.0	
Age [y]	46.0 ± 10.1	47.0 ± 10.1		47.8 ± 8.5	48.9 ± 8.2	
Height [cm]	171.2 ± 9.9	170.8 ± 9.7		170.5 ± 9.1	169.8 ± 8.6	
Weight [kg]	155.7 ± 31.7	107.6 ± 25.8	<0.001	152.1 ± 26.2	105.4 ± 19.7	<0.001
BMI [kg/m^2^]	52.9 ± 8.3	36.8 ± 7.3	<0.001	52.3 ± 8.1	36.6 ± 6.2	<0.001
HbA1c [%]	7.0 ± 1.5	5.7 ± 0.9	<0.001	7.6 ± 1.6	6.0 ± 1.0	<0.001
Insulin use [%]	22.6	0	<0.001	35.8	0	<0.001
Glucose [mg/dl]	136.2 ± 64.0	100.2 ± 34.4	<0.001	161.5 ± 69.6	108.2 ± 40.2	0.011
Triglycerides [mg/dl]	215.3 ± 131.4	157.3 ± 87.4	<0.001	239.3 ± 153.0	177.2 ± 99.1	<0.001
Cholesterol [mg/dl]	185.6 ± 38.1	183.2 ± 41.8	<0.001	185.0 ± 42.7	183.9 ± 45.8	<0.001
HDL [mg/dl]	49.1 ± 43.8	52.0 ± 13.2	0.039	51.4 ± 54.3	50.2 ± 13.4	0.114
LDL [mg/dl]	101.9 ± 34.1	99.4 ± 36.1	<0.001	98.0 ± 39.0	97.7 ± 40.6	<0.001
ASAT [U/l]	27.5 ± 14.4	19.1 ± 7.4	0.029	29.5 ± 15.1	19.0 ± 7.9	0.127
ALAT [U/l]	38.2 ± 18.9	23.9 ± 14.3	0.066	40.0 ± 20.0	24.1 ± 17.1	0.299
GGT [U/l]	54.3 ± 67.7	27.6 ± 23.1	<0.001	59.4 ± 78.8	29.7 ± 27.5	<0.001
Creatinine [mg/dl]	0.90 ± 0.3	0.87 ± 0.26	<0.001	0.93 ± 0.3	0.91 ± 0.29	<0.001
GFR [ml/min]	76.8 ± 22.3	81.1 ± 25.2	<0.001	71.6 ± 21.4	73.4 ± 23.6	<0.001

**Fig. 2:**
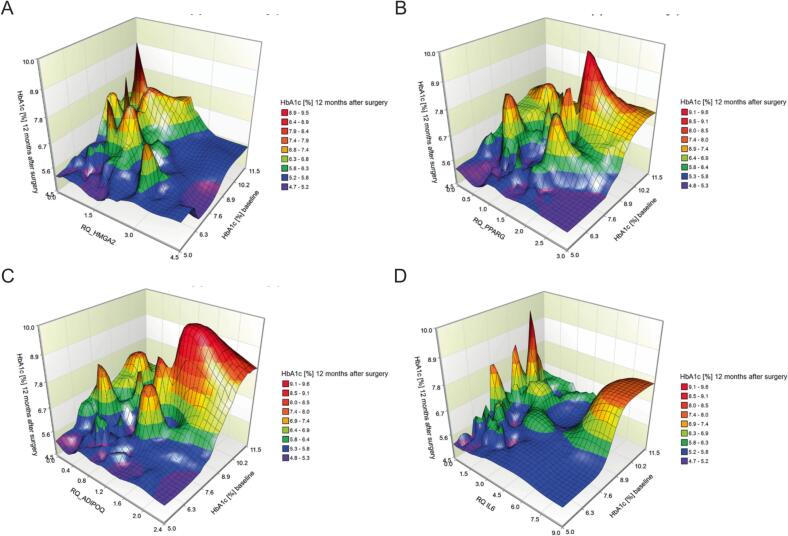
Surface plots depicting the relationship between baseline HbA1c, HbA1c 12 months after surgery and *HMGA2* (A), *PPARG* (B), *ADIPOQ* (C) and *IL6* (D) expression.

**Table 2:** Remission rates for patients with T2DM and prediabetes.

		Diabetes remission		
		No remission [%]	Partial remission [%]	Complete remission [%]
Preoperative status	Diabetes	22.6	24.2	53.2
	Prediabetes		12.8	87.2
Total		13.9	19.8	66.3

We then examined whether the expression of *HMGA2*, *PPARG*, *ADIPOQ* and *IL6* was associated with diabetes remission. To obtain an initial overview, we used 3D surface plots illustrating the functional relationships between preoperative HbA1c, HMGA2 expression, and HbA1c concentrations 12 months after surgery, suggesting that postoperative HbA1c depends on both preoperative HbA1c levels and HMGA2 expression (Fig. 2A). The HbA1c concentration after 12 months depends on preoperative HbA1c values and *HMGA2* expression. If the preoperative HbA1c is above 6.4 % the mean value of HbA1c after 12 months is 6.3 ± 1.0 % for patients with relative *HMGA2* expression below 2.1 and 5.5 ± 0.4 for patients with a relative *HMGA2* expression above 2.1. On average, lower *HMGA2* expression is associated with higher HbA1c levels at 12 months after surgery. We observed that the functional relationship between HbA1c concentration at 12 months, initial HbA1c concentration, *PPARG* expression, and *ADIPOQ* expression showed similar patterns. Higher expression of either *PPARG* or *ADIPOQ* was associated with higher initial HbA1c levels as well as higher HbA1c levels 12 months after surgery. (Fig. 2B, 2C). We also observed that *PPARG* and *ADIPOQ* expression correlated well with each other: *PPARG* = 0.33 + 1.05*ADIPOQ (Sup. Fig. 3 and Table 1). We did not detect a clear pattern for the relationship of *IL6* expression and initial HbA1c with HbA1c 12 months after surgery. Low and high *IL6* expression were associated with higher HbA1c concentrations 12 months after surgery, whereas medium expression was associated with lower HbA1c concentrations (Fig. 2D).

**Fig. 3:**
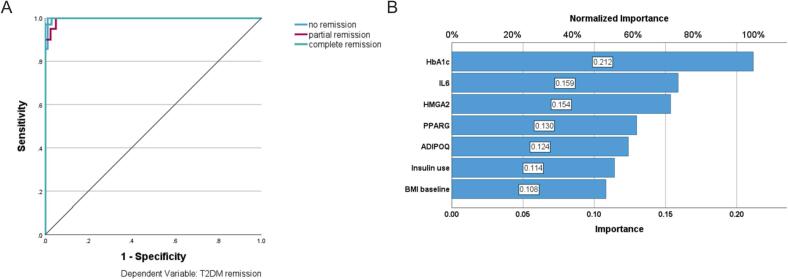
Multilayer perceptron: Receiver operating characteristic curve (ROC), one curve for each type of remission and area under each curve (AUC). AUC: no remission 0.998; partial remission 0.996 and complete remission 0.999 (A) and importance and normalized importance for each predictor (result of a sensitivity analysis). (B).

After a first overview of the functional relationship for each gene and the main outcome parameter, we wondered whether the combination of these gene expressions could more accurately predict the HbA1c concentrations 12 months after surgery. We calculated two multilayer perceptrons, each with two hidden layers. In the first (basic) model we used preoperative HbA1c and BMI as well as insulin dependence as predictors. In the second model (DiaBar model) we added the expressions of *HMGA2*, *PPARG*, *ADIPOQ* and *IL6*. For the two models we took no, partial and complete remission 12 months after surgery as outcome. We obtained an overall accuracy of 82.2 % for the basic model (remission: no 92.9 %; partial 35.0 %; complete 94.0). The DiaBar model with the four biomarkers in addition had an overall accuracy of 98.0 % (remission: no 100 %; partial 90.0 %; complete 100 %) (Table 3). 104 of the 106 patients were correctly classified (Table 3). ROC curves provided further insight into the diagnostic performance of the DiaBar model. The area under the curve (AUC) values were 0.998 for no remission, 0.996 for partial remission and 0.999 for complete remission, indicating nearly perfect discrimination between these classes. In other words, the DiaBar model achieved near-perfect sensitivity and specificity for all T2D remission states (Fig. 3A).

**Table 3:** Multilayer perceptron: classification results.

Classification (DiaBar model)				
	Predicted			
	No remission	Partial remission	Complete remission	Percent Correct
No remission	14	0	0	100 %
Partial remission	1	18	1	90.0 %
Complete remission	0	0	67	100 %
Overall Percent	14.9 %	17.8 %	67.3 %	98.0 %
Dependent Variable: T2DM remission 12 months after surgery				

To understand the value of each predictor we calculated the importance, which measures how much the predicted value of the DiaBar model changes for diverse values of the predictor. We transformed the importance of each predictor into normalized importance in percentage (Fig. 3B).

**Fig. 4:**
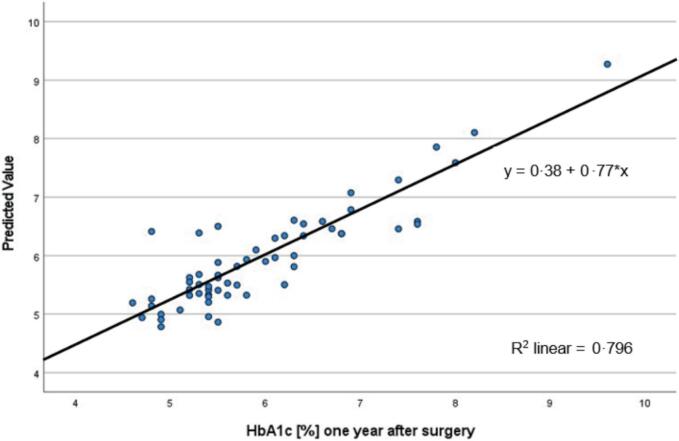
Multilayer Perceptron Network: Predicted versus actual values of HbA1c 12 months after surgery with linear regression for patients with preoperative T2D. R^2^ linear is a measure of the goodness of fit of the model.

Furthermore, another multilayer perceptron model explains 80 % (R^2^ = 0.796) of the variation in predicted 12-month HbA1c levels. This indicates strong performance by the MLP model in predicting HbA1c concentrations at 12-month after surgery. (Fig. 4).

**Fig. 5:**
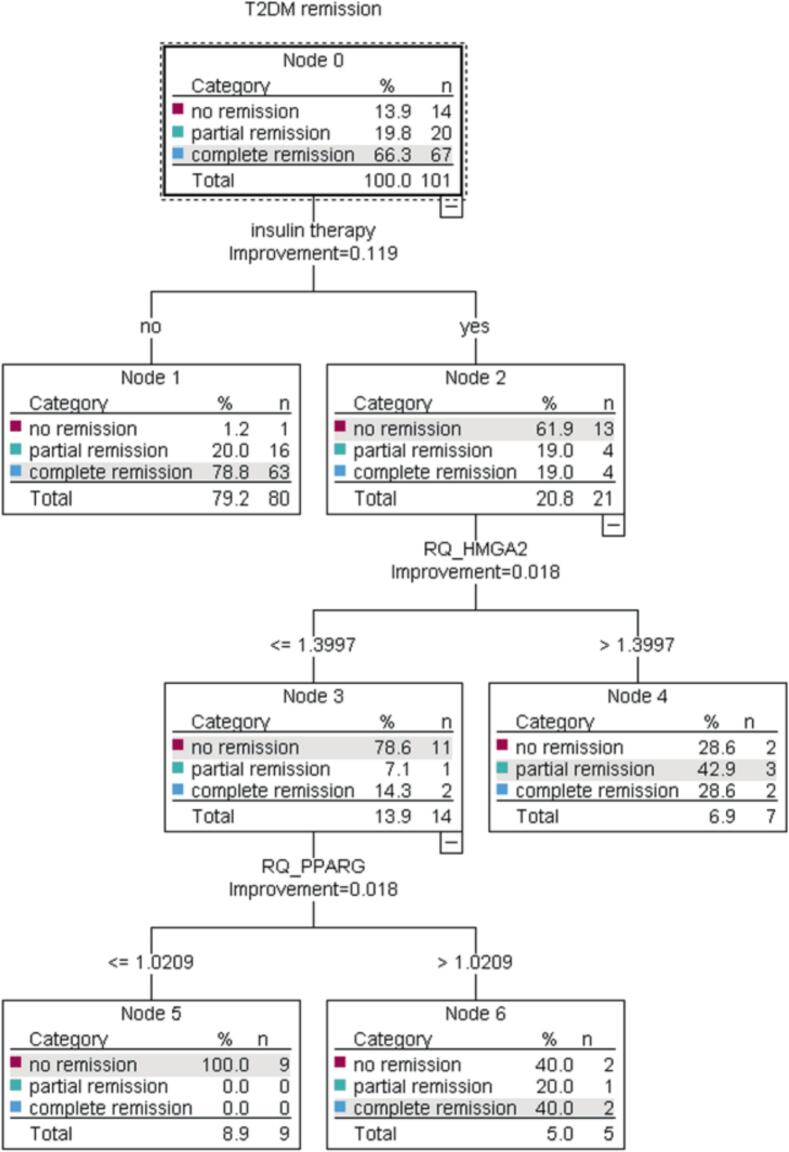
Classification and Regression Tree (CRT) splits the data into segments that are as homogeneous as possible with respect to the dependent variable remission.

Studies have shown that preoperative insulin use is linked to lower rates of diabetes remission^11^. In our data, 98.8 % of patients who weren't on insulin achieved partial or complete remission whereas 61.9 % on insulin therapy had no remission. Accurately predicting remission rates for those on insulin therapy remains a challenge. However, with the above models and biomarkers, we are able to improve prediction for this patient subgroup. For example, 79 % of the insulin-dependent patients with *HMGA2* expression ≤ 1.4 had no remission whereas 71.4 % of the patients on insulin therapy with *HMGA2* > 1.4 had a partial or complete remission at 12 months (Fig. 5).

Finally, we wanted to compare our results with the DiaRem score, one of the most popular, internally and, externally validated score to guide the joint decision-making process between clinicians and patients. 98.7 % of the patients with DiaRem score ≤ 8 and 43.5 % of the patients with DiaRem score > 8 had a partial or complete remission. The overall accuracy of the DiaRem score is 74.3 % (remission: no 92.9 %; partial 0 % and complete 92.5 %) (Table 4). The insulin dependency at baseline had an overall accuracy of 75.2. Adding *HMGA2*, *PPARG*, *ADIPOQ* and *IL6* to the DiaRem score with cut-off value 8 the overall accuracy increases to 96.0 %. Finally, the DiaBar model had an accuracy of 98.0 % (remission: no 100 %; partial 90.0 % and complete 100 %) (Table 4).

**Table 4:** Percentage of no, partial and complete T2D remission for the DiaRem score with cutoff value 8, insulin dependency, DiaRem score with HMGA2, PPAR-γ, ADIPOQ and IL-6 and finally for the DiaBar model with percentages of correct classifications.

Remission 12 months after surgery	DiaRem Score			insulin use			DiaRem score + HMGA2, PPAR-γ, ADIPOQ and IL-6	DiaBar model
	≤8	>8	percent correct	no	yes	percent correct	percent correct	
No remission	1.3	56.5	92.9	1.2	62.0	92.9	92.9	100.0
Partial remission	19.2	21.7	0.0	20.0	19.0	0.0	95.0	90.0
Complete remission	79.5	21.7	92.5	78.8	19.0	94.0	97.0	100
Overall percentage	**77.0**	**23.0**	**74.3**	**79.2**	**20.8**	**75.2**	**96.0**	**98.0**

## Discussion

This pilot study was conducted to analyze the impact of gene expression of *HMGA2*, *PPARG*, *ADIPOQ* and *IL6* on T2D remission after metabolic surgery. Artificial neural networks allowing non-linear correlations were trained to discriminate patients with complete, partial and no diabetes remission after surgery, and the importance of each clinical and genetic parameter was evaluated.

In the first model we used preoperative HbA1c and insulin use as predictors and in the second model (DiaBar) in addition the gene expression of *HMGA2*, *PPARG*, *ADIPOQ* and *IL6*. Interestingly, the basic model with three clinical parameters achieved a higher overall accuracy (82 %) compared to the well-established DiaRem score (74 %). When we compared the DiaBar model with the four additional biomarkers to the basic model, we observed that the overall accuracy jumped from 82 % to 98 %. In DiaBar the preoperative HbA1c concentration was the most important predictor of diabetes remission, which was also supported by others [11,30]. The second-best predictor was *IL6* followed by *HMGA2* expression. *HMGA2* was found to be upregulated in the SAT of patients with T2D. We observed that higher expression of *HMGA2* was associated with lower HbA1c 12 months after surgery. A possible explanation for this observation is that BMI is also associated with HMGA2 expression and a lower BMI is associated with higher HbA1c concentrations after surgery. Thus, higher *HMGA2* expression leads to lower HbA1c levels. *PPARG* and *ADIPOQ* expression were the next best predictors in our model, followed by insulin use and finally by BMI before surgery. High expression of *ADIPOQ* and *PPARG* were associated with both high preoperative HbA1c and high postoperative HbA1c. Insulin use has also been shown by others to be an important factor [11].

With DiaBar, we have developed a new approach to predict outcome after metabolic surgery by profiling subcutaneous adipose tissue. Subcutaneous adipose tissue can be easily sampled during surgery in a large number of patients. However, in order to facilitate informed decisions prior to surgery, new ways of sampling SAT need to be developed. In addition, with this study, we wanted to provide proof of principle that SAT can be used to more accurately predict diabetes remission after metabolic surgery.

We acknowledge that this retrospective pilot study has several limitations. First, we did not include patients without diabetes because our primary comparison focuses on patients with diabetes categorized into complete remission, partial remission, or no remission; individuals without diabetes would not fit into these groups. Second, although the sample size of 106 patients is relatively small, the highly significant results suggest that the study was adequately powered. Furthermore, the fact that the study achieves such highly significant results already with small numbers emphasizes, together with Cohen’s d, that the relevance (effect) of our hypothesis is substantial. A key limitation of our study is the relatively short follow-up period, as postoperative hemoglobin A1c levels were assessed only up to 12 months. Longer-term studies are needed to evaluate the sustained efficacy of the surgical intervention, given that metabolic improvements may decline over time. Another limitation of our study is that we did not perform a formal cost-effectiveness analysis comparing our predictive approach with existing tests, which would be essential to fully assess its clinical and economic viability. Another potential limitation of our approach is the reliance on intraoperative subcutaneous adipose tissue biopsies and gene expression profiling, which may restrict widespread clinical use due to logistical and cost-related challenges. We aim to address this in future studies by investigating the feasibility of preoperative sampling and analysis to enhance the practical applicability of our predictive tool. Finally, we simultaneously compared patients undergoing sleeve gastrectomy with those undergoing RYGB. Although previous reports have indicated a higher rate of diabetes remission following RYGB, no significant differences were observed in our study at the endpoint. Overall, our next goal is to generate a larger data set with external validation to confirm our findings and improve the sampling method.

## Conclusions

In conclusion, the newly developed DiaBar test has the potential to accurately predict diabetes remission in patients undergoing metabolic surgery. The accuracy appears to be superior to the DiaRem score. However, further research is warranted to confirm our findings and to improve the sampling method.

## Availability of data and materials

The datasets used and/or analysed during the current study are available from the corresponding author on reasonable request.

## CRediT authorship contribution statement

**Jonas Wagner:** Writing – review & editing, Writing – original draft, Validation, Software, Methodology, Investigation, Formal analysis, Data curation, Conceptualization. **Manfred Wischnewsky:** Writing – review & editing, Writing – original draft, Visualization, Validation, Software, Resources, Project administration, Methodology, Formal analysis, Data curation, Conceptualization. **Patricia von Kroge:** Writing – review & editing, Investigation, Data curation. **Helge Wilhelm Thies:** Writing – review & editing, Project administration, Methodology. **Pia Roser:** Writing – review & editing, Supervision, Investigation. **Stefan Wolter:** Writing – review & editing, Supervision, Methodology, Funding acquisition. **Thilo Hackert:** Writing – review & editing, Supervision, Resources, Project administration. **Jakob Izbicki:** Writing – review & editing, Supervision, Resources, Project administration, Funding acquisition. **Oliver Mann:** Writing – review & editing, Supervision, Resources, Project administration, Funding acquisition, Formal analysis, Conceptualization. **Anna Duprée:** Writing – review & editing, Supervision, Project administration, Funding acquisition, Formal analysis, Conceptualization.

## Ethics approval and consent to participate

All procedures performed in studies involving human participants were in accordance with the ethical standards of the institutional and/or national research committee and with the 1964 Helsinki Declaration and its later amendments or comparable ethical standards. Our institutional review board (Ethik-Kommission der Ärztekammer Hamburg) approved our biobank and study (PV4889). Informed consent was obtained from all individual participants included in the study.

## Funding

Funding was provided by Lipocyte BioMed GmbH. The sponsor had no role in the design and conduct of the study, data collection, data management, data analysis, and data interpretation; preparation, review, or approval of the manuscript, and decision to submit the manuscript for publication. The corresponding author had full access to the data.

## Declaration of competing interest

The authors declare the following financial interests/personal relationships which may be considered as potential competing interests: Oliver Mann reports financial support and equipment, drugs, or supplies were provided by Lipocyte BioMed GmbH, Erbrichterweg 7a, 28,357 Bremen, Germany. Helge Wilhelm Thies reports a relationship with Lipocyte BioMed GmbH, Erbrichterweg 7a, 28,357 Bremen, Germany that includes: employment. Lipocyte BioMed GmbH holds patents related to this study. If there are other authors, they declare that they have no known competing financial interests or personal relationships that could have appeared to influence the work reported in this paper.
