# Supplementary material for: DiaBar: Predicting type 2 diabetes remission post-metabolic surgery utilizing mRNA expression profiles from subcutaneous adipose tissue
Source: J Clin Transl Endocrinol. 2025 Jul 22;41:100410. doi: 10.1016/j.jcte.2025.100410 (PMC12309261; doi:10.1016/j.jcte.2025.100410)
Supplement: Supplementary Data 1 [file mmc1.docx]

**Supplement**

Supplementary Figure 1 The decrease in BMI over 12 months after sleeve gastrectomy or RYGB surgery is depicted. There is no significant (p=0.370) difference between sleeve gastrectomy and RYGB on BMI reduction after 12 months by adjusting for BMI baseline.

Supplementary Figure 2 The decrease in HbA1c over 12 months after sleeve gastrectomy or RYGB surgery is depicted. HbA1c improves highly significantly over the period of 12 months (p <0.001) for both types of operation, but the difference between sleeve gastrectomy and RYGB over time is not significant (p=0.643).

Supplementary Figure 3 Curve fitting: An appropriate mathematical model that expresses the relationship between the dependent variable PPARG and the independent variable ADIPOQ and estimates the values of its parameters with 95% confidence limits using nonlinear regression.

Supplementary Table S1: Primer sequences for preamplification step

| **Primer** | **Sequence (5’ -> 3’)** |
| --- | --- |
| IL-6 206F | ACC CCC AGG AGA AGA TTC CA |
| IL-6 367R | GCC TCT TTG CTG CTT TCA CA |
| HMGA2 899F | GGC CGC CCC AGG AA |
| HMGA2 965R | GTC TTC CCC TGG GTC TCT TAG G |
| HPRT FP | GGC AGT ATA ATC CAA AGA TGG TCA A |
| HPRT RP | GTC TGG CTT ATA TCC AAC ACT TCG T |

Supplementary Table S2 The values of the parameters A and B with 95% confidence limits.

| **Parameter** | **Parameter** | **Asymptotic** | **Lower** | **Upper** |
| --- | --- | --- | --- | --- |
| **Name** | Estimate | Standard Error | 95% C.L. | 95% C.L. |
| **A** | 0.33 | 0.07 | 0.20 | 0.46 |
| **B** | 1.05 | 0.07 | 0.91 | 1.19 |

Supplementary Table S3 DiaBar model: multilayer perceptron network information.

| **Network information** | |  |  |
| --- | --- | --- | --- |
| Input Layer | Factors | 1 | **Insulin (no, yes)** |
|  | Covariates | 1 | **Preoperative HbA1c [%]** |
|  |  | 2 | **RQ *HMGA2*** |
|  |  | 3 | **RQ *PPARG*** |
|  |  | 4 | **RQ *ADIPOQ*** |
|  |  | 5 | **RQ *IL6*** |
|  |  | 6 | **Preoperative BMI** |
|  | Number of Unit ins Input Layer*a | | 8 |
|  | Rescaling Method for Covariates | | Standardized |
| Hidden Layer | Number of Hidden Layers | | 2 |
|  | Number of Units in Hidden Layer 1 | | 7 |
|  | Number of Units in Hidden Layer 2 | | 5 |
|  | Activation Function | | Hyperbolic tangent |
| Output Layer | Dependent Variables | | T2D Remission (no, complete, partial) |
|  | Number of Units | | 3 |
|  | Activation Function | | Softmax |
|  | Error Function | | Cross-entropy |
| *a Excluding the bias unit; the factor insulin is represented by 2 imput units | | | |
