# Supplementary figures and images for: DiaBar: Predicting type 2 diabetes remission post-metabolic surgery utilizing mRNA expression profiles from subcutaneous adipose tissue
Source: J Clin Transl Endocrinol. 2025 Jul 22;41:100410. doi: 10.1016/j.jcte.2025.100410 (PMC12309261; doi:10.1016/j.jcte.2025.100410)

Means of BMI

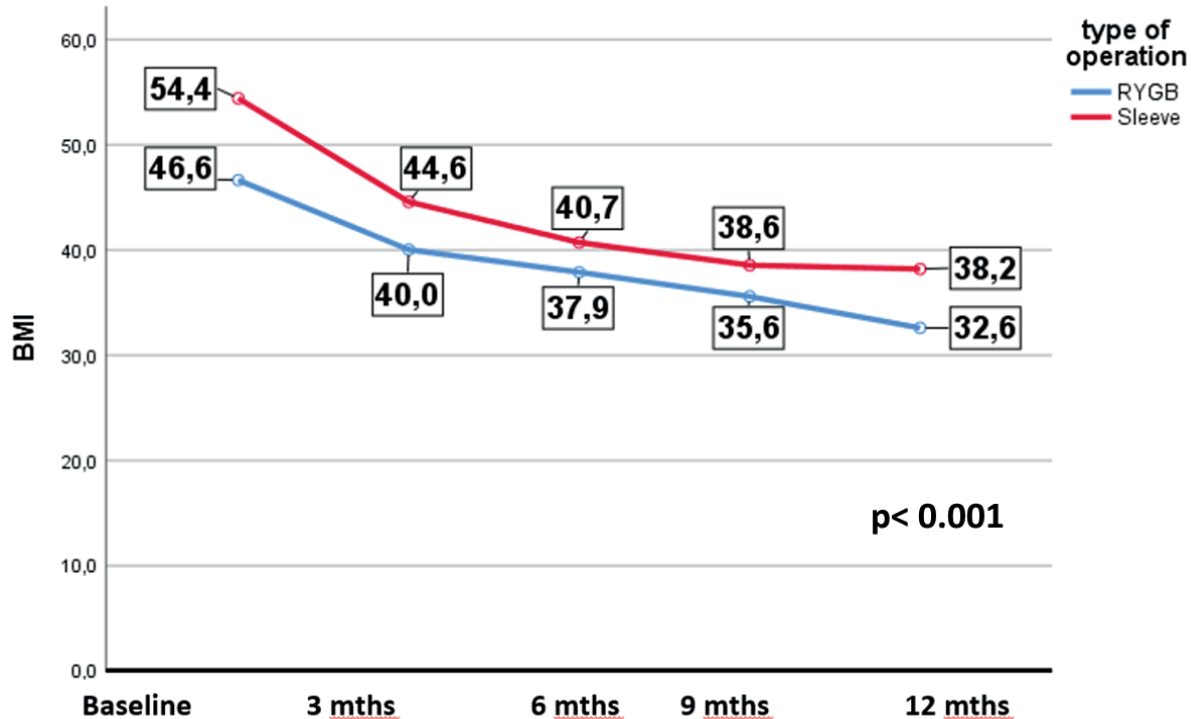

Supplement: Supplementary Data 2 [file mmc2.pdf]

## HbA1c of RYGB vs Sleeve over the period of 12 months

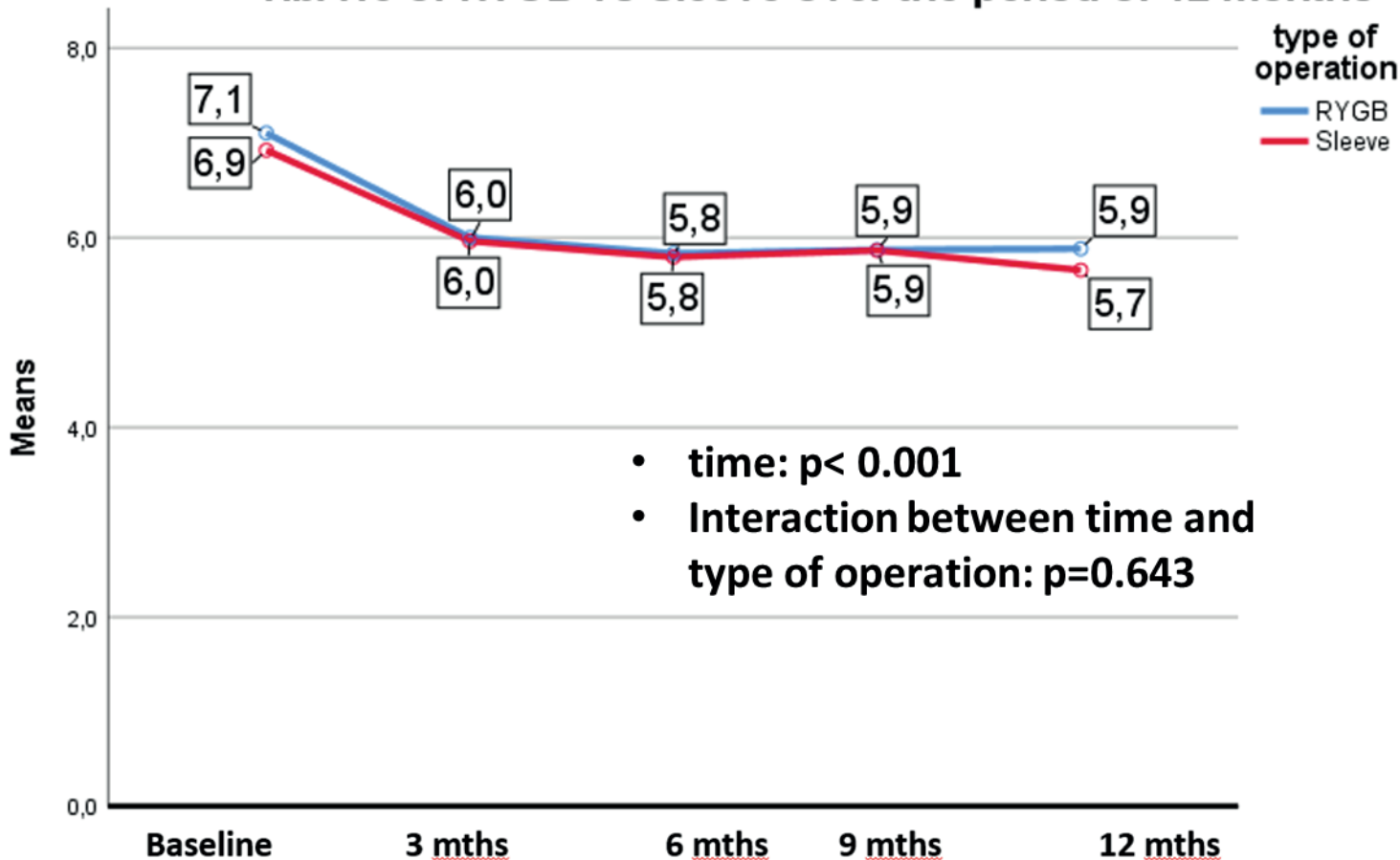

Supplement: Supplementary Data 3 [file mmc3.pdf]

# PPARG vs ADIPOQ

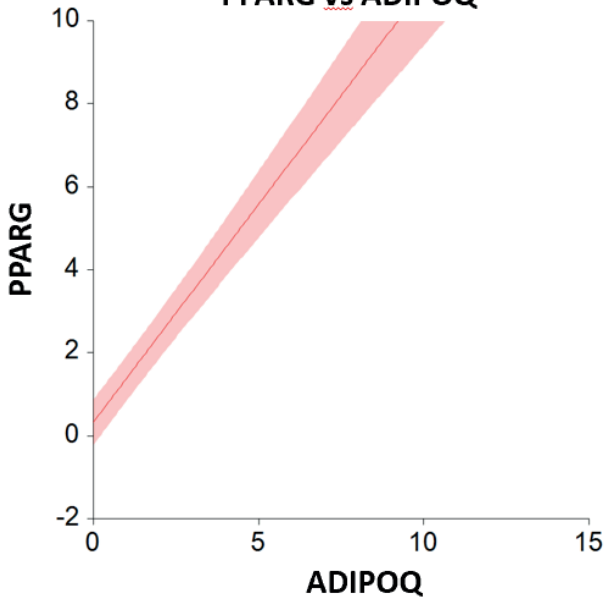

Model:  $PPARG = A + B \cdot ADIPOQ$

Supplement: Supplementary Data 4 [file mmc4.pdf]
